# SARS-CoV-2 Vaccines Based on the Spike Glycoprotein and Implications of New Viral Variants

**DOI:** 10.3389/fimmu.2021.701501

**Published:** 2021-07-12

**Authors:** Daniel Martínez-Flores, Jesús Zepeda-Cervantes, Adolfo Cruz-Reséndiz, Sergio Aguirre-Sampieri, Alicia Sampieri, Luis Vaca

**Affiliations:** ^1^ Departamento de Biología Celular y del Desarrollo, Instituto de Fisiología Celular, Universidad Nacional Autónoma de México, Mexico City, Mexico; ^2^ Departamento de Microbiología e Inmunología, Facultad de Medicina Veterinaria y Zootecnia, Universidad Nacional Autónoma de México, Mexico City, Mexico; ^3^ Laboratorio de Fisicoquímica e Ingeniería de Proteínas, Facultad de Medicina, Universidad Nacional Autónoma de México, Mexico City, Mexico

**Keywords:** spike glycoprotein, RBD, vaccine design, SARS-CoV-2 variants, resistance to neutralization

## Abstract

Coronavirus 19 Disease (COVID-19) originating in the province of Wuhan, China in 2019, is caused by the severe acute respiratory syndrome coronavirus type 2 (SARS-CoV-2), whose infection in humans causes mild or severe clinical manifestations that mainly affect the respiratory system. So far, the COVID-19 has caused more than 2 million deaths worldwide. SARS-CoV-2 contains the Spike (S) glycoprotein on its surface, which is the main target for current vaccine development because antibodies directed against this protein can neutralize the infection. Companies and academic institutions have developed vaccines based on the S glycoprotein, as well as its antigenic domains and epitopes, which have been proven effective in generating neutralizing antibodies. However, the emergence of new SARS-CoV-2 variants could affect the effectiveness of vaccines. Here, we review the different types of vaccines designed and developed against SARS-CoV-2, placing emphasis on whether they are based on the complete S glycoprotein, its antigenic domains such as the receptor-binding domain (RBD) or short epitopes within the S glycoprotein. We also review and discuss the possible effectiveness of these vaccines against emerging SARS-CoV-2 variants.

## Introduction

COVID-19 (Coronavirus Disease 19) was the name of a new outbreak of several cases of pneumonia occurred in Wuhan (Hubei province, China) in December 2019 (1). Soon after, the virus was isolated and associated with a severe acute respiratory syndrome coronavirus (SARS-CoV), and for this reason named as SARS-CoV-2. Like SARS-CoV-1 and MERS-CoV (Middle East respiratory syndrome coronavirus), this virus is mainly transmitted through aerosols in poorly ventilated areas (2). When the infections began to grow rapidly and even with the call for confinement, the levels of transmission and severity of COVID-19 became evident, and for this reason COVID-19 was declared a pandemic on March 11, 2020 (3). So far, more than 2 million deaths have occurred, highlighting the need for the rapid development of vaccines.

The coronavirus receive their name because of the “Corona” spikes produced by the S glycoprotein protruding the viral capsid. This protein is responsible for anchoring to the host receptor, the angiotensin-converting enzyme 2 (ACE2).

S glycoprotein is responsible for the entry of the virus into host cells, where it begins to spread, but it can also be recognized by the immune system triggering a protective response, the main objective of vaccines (4, 5). Several types of new vaccines currently in use are selected based on their ability to generate neutralizing antibodies upon immunization (6–9).

The development and testing of vaccines by the scientific and medical communities for SARS-CoV-2 has been extraordinary. The first approved vaccines were based on mRNA and have been applied in several countries (10, 11). Adenovirus vector-based vaccines and vaccines containing inactivated virus followed, and nanoparticle-based vaccines using the baculovirus system would likely be approved soon. There are high expectations for all the vaccines, but we do not know yet how long the vaccines will protect against SARS-CoV-2 and if they will also protect against the newly emerging genetic variants.

It is important to remember that virus infection is not always protective, for this reason, there are multiple cases of reinfection (12, 13). In many cases reported so far after infection the levels of antibodies decrease rapidly without a clear explanation for these results. For instance, IgM against SARS-CoV-2 rapidly declines until it becomes undetectable, whereas IgG remains for at least 6 months in symptomatic COVID-19 patients (14). Although the immune response has been shown to have high variability, the cellular immune response based on long-lasting T-cell immunity seems to play an important role in the control of SARS-CoV-2 infection (15).

The appearance of several highly transmissible SARS-CoV-2 variants is the new challenge for current vaccines. SARS-CoV-2 mutations occur frequently, these mutants are not considered new strains, but only variants, and they represent a concern regarding the effectiveness of current vaccines. It has been estimated that 1-2 single nucleotide mutations per month accumulate in SARS-CoV-2 variants (16). So far approved vaccines protect against infection, but the question remains about the effectiveness of currently used vaccines against the new variants. New trials underway inspired by the molecular study of epitope-antibody interactions may shed light into the effectiveness of current vaccines against novel variants. In this review, we focused on the vaccine development against SARS-CoV-2, highlighting the different approaches using full-length S glycoprotein, RBD and epitopes from the S glycoprotein, and discussed their advantages and drawbacks as potential antigens. We analyzed some platforms for vaccine manufacturing and discussed its effective selection.

## Understanding the Biology of SARS-CoV-2 to Develop a Vaccine

### SARS-CoV-2 From the Coronavirus Family

The Coronaviridae family is composed of enveloped viruses of approximately 65-125 nm in diameter, they contain a single-stranded positive RNA (ssRNA) segment with 26-32 kb in length. This virus has been classified into 4 genera: Alpha, Beta, Gamma and Delta-coronavirus (17). Seven viruses of this family affect humans, and most of them belong to the alphacoronavirus and betacoronavirus genus (17).

Betacoronaviruses have been recognized as emerging zoonotic viruses with the potential to generate pandemics (18). SARS-CoV-1 emerged in southern China in 2002 and was related to bat coronaviruses. SARS-CoV-1 is thought to have adapted to reservoir animals such as palm civets and raccoon and eventually adapt to humans (19, 20). In 2012, MERS-CoV emerged in Saudi Arabia, it was identified as originated from bats as well, but infected camels likely caused zoonosis into humans (21). A third Betacoronavirus, SARS-CoV-2 emerged in China 8 years later in 2019, causing one of the biggest pandemics in recent times, whose origin is also related to bats (17). SARS-CoV-2 shares an ~80% nucleotide sequence identity with the SARS-CoV-1, and 54% with MERS-CoV (22).

SARS-CoV-1 spread to more than 37 countries and reached a case-fatality rate of 15%, but since 2004 no cases have been reported. MERS was distributed in 27 countries reaching a fatality rate of 34% with sporadic outbreaks until 2016 (23). On the other hand, it has been reported that SARS-CoV-2 has a fatality rate between <1 to 9% or in some cases higher depending on the age of the affected population and comorbidities (24).

### SARS-CoV-2 Structure

**Figure 1:**
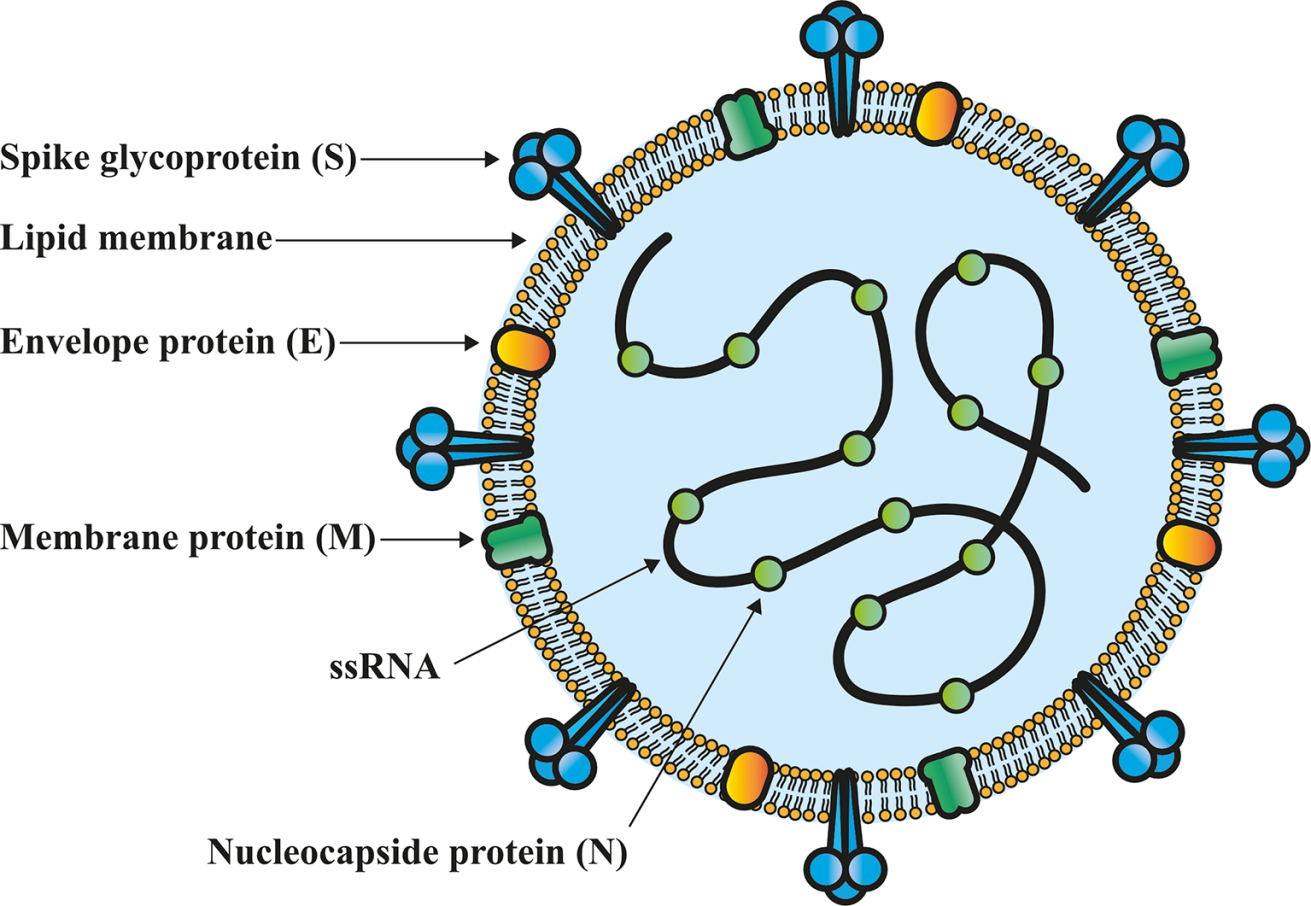
Structural characteristics of SARS-CoV-2 virion. Nucleocapsid (N) protein is associated to the single-stranded genomic RNA (ssRNA), which is covered by an outer envelope of the main structural proteins: S glycoprotein (S), membrane protein (M) and envelope protein (E), which are found in the lipid membrane of the virion.

The coronaviruses have a genome size of approximately 26-32 kb, which encodes for the structural proteins: S, a glycoprotein that forms trimers on the viral surface and is essential for entry into the target cell (5); the envelope (E) protein that participates in the morphogenesis and assembly of virions (25), while membrane (M) and nucleocapsid (N) proteins play a fundamental role in viral RNA packaging (26) (**Figure 1**). On the other hand, the sequences encoding for 16 non-structural proteins (Nsp1-16) have been identified. Nsp1, Nsp4, Nsp7-9, Nsp12 and Nsp13 have the main function of participating in the viral replication-transcription. Nsp2, participates in the modulation of host cell survival signaling pathways. Nsp3 and Nsp5 play a role in the cleavage of the viral polyprotein. Nsp6, induces the formation of autophagosomes into the endoplasmic reticulum of host cells. Nsp10, Nsp14 and Nsp16 have exoribonuclease function and a role in mRNAs cap methylation. Nsp15 has endoribonuclease activity while Nsp11 function remains unknown (27).

**Figure 2:**
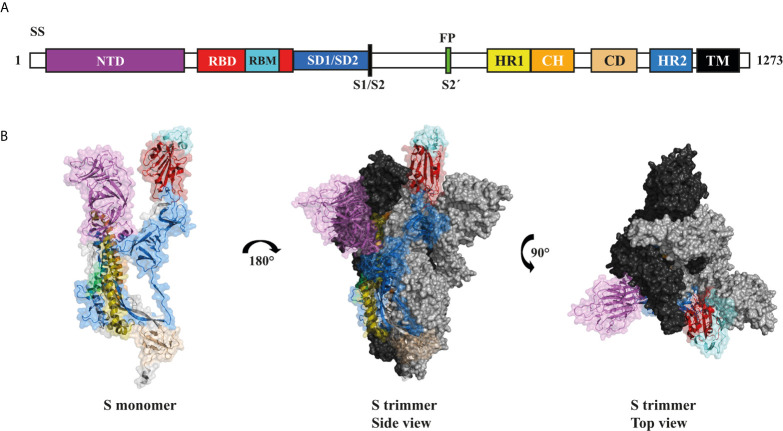
Structural characteristics of the trimeric S glycoprotein of SARS-CoV-2. **(A)** Schematic representation of the composition and arrangement of domains present in the SARS-CoV-2 S glycoprotein. Signal sequence (SS), N-terminal domain (NTD), receptor-binding domain (RBD), subdomain 1 and 2 (SD1/2), furin cleavage site (S1/S2, arrow), protease cleavage site 2′ (S2′, arrow), fusion peptide (FP), heptad repeat 1 (HR1), central helix (CH), connector domain (CD), heptad repeat 2 (HR2), transmembrane domain (TM), cytoplasmic tail (CT). **(B)** Side view of the SARS-CoV-2 S glycoprotein monomer (PDB: 7DK3) in open state using the same color representation as in panel A (left). Side (middle) and top (right) views of the oligomeric conformation. The second (grey) and third (black) sub-units of the trimer are shown in the closed conformation.

The viral S glycoprotein is in a metastable prefusion state, through the association of subunits 1 and 2 (S1 and S2) *via* non-covalent interactions (28). The S1 subunit of S is made up of 672 amino acids (residues 14-1273) and contains four domains: an N-terminal domain (NTD), the RBD, and the subdomains 1 and 2 (SD1 and SD2) (28). RBD has received more attention because it is recognized as the intermediary factor in the virus-host cell interaction, through the interaction of its receptor-binding motif (RBM) with the angiotensin converting enzyme 2 (ACE2) of the host cell. The binding of RBM to the ACE2 receptor is crucial in the viral infection process, since it has been shown that this interaction induces the transition of S from a metastable prefusion state to a more stable post-fusion state, which is required for membrane fusion between the virus and the host cell. The S2 subunit is composed of 588 amino acids (residues 686-1273), contains an N-terminal fusion peptide (FP) and two heptad repeats (HR1 and HR2) that mediate the association of the S2 subunit to the host membrane (29, 30). The S2 subunit also have a transmembrane domain (TM) and a cytoplasmic tail (CT) which serves to attach the S glycoprotein to the virus membrane (28) (**Figure 2**).

SARS-CoV-2 uses S glycoprotein which has high affinity for the ACE2 receptor, to attach to the host cells, similarly to SARS-CoV-1. Cathepsin B and L, furin as well as transmembrane protease serine 2 (TMPRSS2) enable the viral entry through cleavage of S glycoprotein in S1 and S2, which are essential for cell binding and membrane fusion, respectively (31). Inhibitors of these proteases block the virus entry (2). Owing to the high homology between SARS-CoV-1 and SARS-CoV-2, they share some antigenic regions. Similarly, MERS-CoV and SARS-CoV-2 share 35% identity on S glycoprotein (32). Most significantly, the RBD of S glycoprotein from SARS-CoV-1 and SARS-CoV-2 share high homology and both are involved in the binding to ACE2 (33).

## Using S Glycoprotein in Coronavirus Vaccines

### SARS-CoV-1 and MERS-CoV Vaccines

Vaccines used against SARS-CoV-1 and MERS-CoV are based on S glycoprotein and induce neutralizing antibodies (nAbs), which are protective (34). Patients recovered from SARS generate antibodies against S glycoprotein, and several studies have confirmed that these vaccines generate protective immunity against SARS-CoV-1 (35). Plasma from influenza and SARS-CoV-1 patients has been shown to decrease the viral load as well as the mortality in patients infected by these viruses (34, 36–40). Similarly, plasma transfusion has been used for the treatment of COVD-19, improving patient prognosis (41).

SARS-CoV-2 vaccines are based on nucleic acids (RNA and DNA), attenuated and inactivated viruses, and many use adjuvants such as aluminum hydroxide. Attenuated virus, replicative viral vectors and no replicative viral vectors based-vaccines (rVV and nrVV) have also been designed and they have been shown to reduce mucosal replication and viral shedding (22).

Nucleic acid-based vaccines can be produced quickly and therefore are a good alternative for rapid responses to pandemics. These include CureVac (mRNA-based), and Inovio (DNA-based). These vaccines need to be administered in lipid nanoparticles, which provide stability and adjuvant effect (42).

An important point to keep in mind during the development of nucleic acid vaccines is that these vaccines require the translocation to the nucleus, where the DNA is transcribed into mRNA. In the case of mRNA vaccines, these processes are not necessary since the mRNA is translated into proteins when it reaches the cytoplasm after the vaccine enters the cells. One of the challenges of the SARS-CoV-2 vaccines is that they should not induce adverse reactions such as antibody-dependent enhancement (ADE) or enhanced respiratory disease as it has been observed with other human and animal coronavirus vaccines (43).

Safety issues for SARS-CoV-1 vaccines generally include: lymphocytic, monocytic, or eosinophilic infiltration of the lung and liver (33). Overall, SARS-CoV-1 based vaccines are protective, but some of them, including whole virus, virus like particles (VLP), or DNA-based vaccines can prompt a Th2 response after vaccination with eosinophilic infiltrate in the lungs, suggesting that these vaccines generate hypersensitivity reactions (44). A Th17 response has also been observed after vaccination with inactivated virus and viral vector-based vaccines. These immunopathologies have been associated with protein N from SARS-CoV-2 as well as with the cytokine storm (overexpression of proinflammatory cytokines: mainly IL-6 and IL-8) (33).

For the adequate design of a SARS-CoV-2 vaccine, any possible immunopathology must be considered. It has been observed that disease caused by SARS-CoV-1 occur at day 3 after the onset of the first symptoms, interestingly when viral load in the respiratory tract decreases (45). Humoral immune response plays a pivotal role during infection. SARS-CoV-1 patients that generate nAbs in the second week of illness, develop a disease more severe than patients who generate nAbs at week 3 (46). The increment in antibody titers during SARS-CoV-1 infection appears to induce acute lung damage leading to death in animal models including macaques (34, 47). In other animal models of infection by coronaviruses (such as feline infectious peritonitis virus), it has been shown that low nAb titers produced after vaccination enhance the disease leading to a higher mortality rate in vaccinated cats (34). This immunopathogenesis can be associated to the process of internalization of virus-antibody complexes by Fc receptors of macrophages leading to ADE as observed in a second dengue virus infection or even after vaccination (48, 49). Another example of ADE is observed with the inactivated respiratory syncytial virus vaccine. ADE may occur because the antigen has been damaged or altered due to the formalin used for inactivation, thus generating non-nAbs and/or inducing a Th17 response (33, 49).

Fortunately, the risk of ADE has already been significantly reduced in some viral vaccines. This has been achieved by including only some protein domains (such as DSV4 containing the EDIII antigen of the 4 dengue serotypes) instead of whole proteins and/or avoiding the inclusion of membrane proteins (prM) (protein related to ADE) such as those used in the design of most appropriated dengue virus vaccines (48). Therefore, although there are no reports that indicate that the COVID-19 is more severe in re-infected patients, the potential for developing ADE after vaccination against SARS-CoV-2 must be avoided. As an attempt to minimize this immunopathology, it has been suggested to use RBD alone (33).

Another important finding was the demonstration that antibodies against SARS-CoV-1 can neutralize SARS-CoV-2 (33, 35). During the onset of the SARS-CoV-2 pandemic it was suggested that a rapid vaccine against SARS-CoV-2 could be generated from SARS-CoV-1 (50). Burton et al. (32) suggested that a suitable vaccine against COVID-19 must possess the following characteristics: ability to induce nAbs, induce high levels of antibodies, production of memory B cells with the ability to differentiate into plasma cells, adequate activation of Fc receptors to provide effector functions that promote a cellular immune response and that does not generate adverse reactions such as “ADE” and “enhanced respiratory disease” (32).

Although some research suggests that antibodies are involved in the worsening of the disease, probably due to ADE, the lesions found in the lungs of people with COVID-19 show lymphocytic infiltrates (34, 51). This observation could indicate that the exacerbated cellular response may be responsible for the immunopathologies associated to the worsening of COVID-19 patients symptoms.

The knowledge and experience that SARS-CoV-1 vaccines have generated during the last years must also be taken into account. These vaccines that include S glycoprotein or RBD are immunogenic and protect against the disease (52). On the other hand, vaccines containing other viral proteins such as protein N without S glycoprotein increase pathology after immunization (53). Interestingly, some SARS vaccines containing S glycoprotein can also increase pulmonary disease (54). Fortunately, it has been demonstrated that antibodies raised against RBD from SARS-CoV-2 neutralize the infection in animal models, so that it represents an excellent vaccine candidate (32). Besides SARS-CoV-1 and SARS-CoV-2 viruses share identical epitopes of T and B cells for structural proteins, the most important being N and S proteins (23). N and S structural proteins of SARS-CoV-1 are the most immunogenic, and the T cell response against these proteins has been reported to be long-lasting (23, 55). It has been observed that 50% of SARS-CoV-1 patients contain CD4^+^ T cells that react to peptides from SARS-CoV-2, suggesting cross immunity with other coronaviruses (56, 57).

An important approach is the so-called “reverse vaccinology 2.0” which consist of identifying epitopes recognized by nAbs from naturally infected patients (58). Another important strategy would be the production of an antigen with native trimeric conformation, reducing the generation of non-nAbs, as occurs with the fusion loops of some flaviviruses (32, 59). A reliable SARS-CoV-2 vaccine should not generate non-nAbs, since they can form immune complexes that are deposited mainly in lung capillaries, activating the complement and leading to the generation of hypersensitivity and tissue damage (32).

### The S Glycoprotein, the Main Target in SARS-CoV-2 Vaccines

Since the S glycoprotein plays an important role in the entry of the virus into host cells, it has been the main target of many vaccines since antibodies against this protein block the entry of the virus, inhibiting viral replication. The sequence of this protein was published on January 10, 2020 (60).

In addition, an antibody that is capable of binding to RBD of both viruses SARS-CoV-1 and SARS-CoV-2 has been identified to neutralize both infections. The binding of this antibody to both RBDs has suggested that this site is less likely to mutate than another sites (32). Therefore, the RBD has been proposed as an excellent vaccine target.

## S Glycoprotein in SARS-Cov-2 Vaccines

It has been shown that the S protein of SARS-CoV-2, is the ideal target for vaccine development on multiple platforms (6, 8, 61) due to its high antigenicity and ability to induce robust immune responses (5, 62, 63). Several reviews have documented and described SARS-CoV-2 vaccines, most based on the development of novel platforms (6), some others have explored the components of these and a few have focused on detailing with the properties of the displayed antigen (61).

In this section, we focus and describe characteristics and design strategies of antigens used in those SARS-CoV-2 vaccines, based on S glycoprotein or its immunogenic regions. We describe in detail the intermolecular modifications to the S glycoprotein, as well as those fusion or conjugation components that can be associated with an improvement in its expression, recognition, stability and/or immunogenicity.

### Full-Length S Glycoprotein Vaccines

SARS-CoV-2 vaccines that use the S glycoprotein seek to preserve its native structural characteristics and induce a robust immune response that protects the individual against possible infections from the original virus (6). Many companies and institutions have chosen to develop SARS-CoV-2 vaccines, using the complete S glycoprotein with and without substantial modifications (**Table 1**).

**Table 1:** Summary of the redesign of the complete S glycoprotein used in vaccines against SARS-CoV-2.

Developers	Vaccine	Vaccine platform	Administration (# doses)	Unfolded S protein design
Vaxart	VXA-CoV2-1	nrVV	Oral (2)	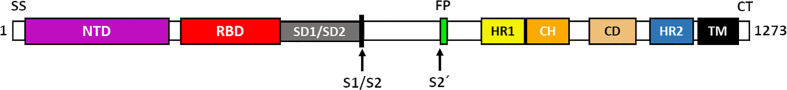
Arcturus Therapeutics	LUNAR-COV-19/ARCT-021	RNA	ND	
AstraZeneca + University of Oxford	Chadox1/AZD1222	nrVV	IM (2)	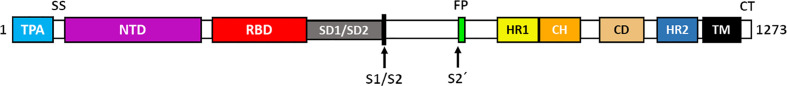
CanSino Biological Inc. + Beijing Institute of Biotechnology	Ad5-nCoV	nrVV	IM (1)	
Genexine Consortium	GX-19	DNA	IM (2)	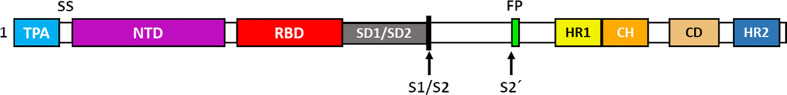
ReiThera + Leukocare + Univercells	GRAd-COV2	nrVV	IM (1)	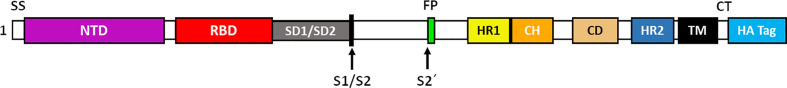
University of Munich (Ludwig-Maximilians)	MVA-SARS-2-S	nrVV	IM (2)	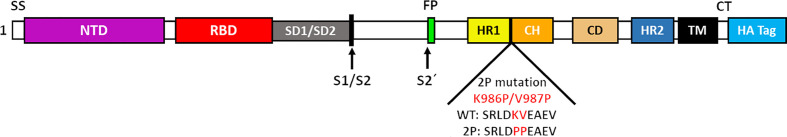
Pfizer-Biontech	BNT162b2	nrVV	IM(2)	
Moderna	mRNA-1273	RNA	IM(2)	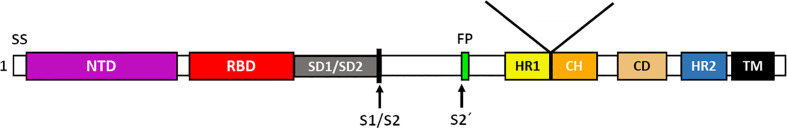
Inovio Pharmaceuticals + International Vaccine Institute + Advaccine (Suzhou) Biopharmaceutical Co., Ltd	INO-4800	DNA	ID (2)	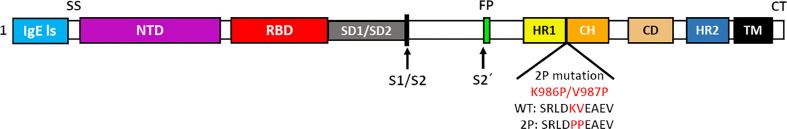
Janssen Pharmaceutical	Ad26.COV2.S	nrVV	IM (2)	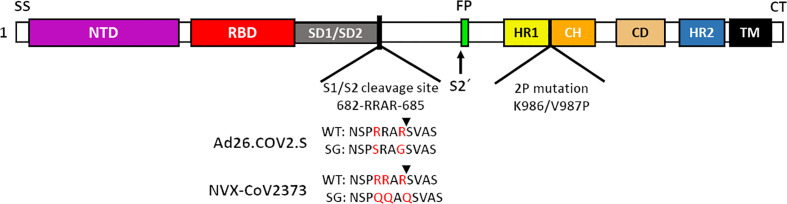
Novavax	NVX-CoV2373	PS	IM (2)	
Clover Biopharmaceuticals Inc. + GSK + Dynavax	SCB-2019	PS	IM (2)	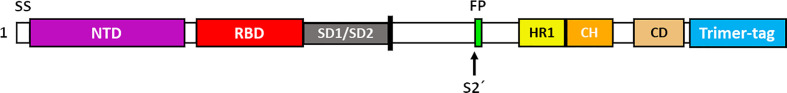
CSL Ltd. + Seqirus + University of Queensland	Sclamp	PS	IM (2)	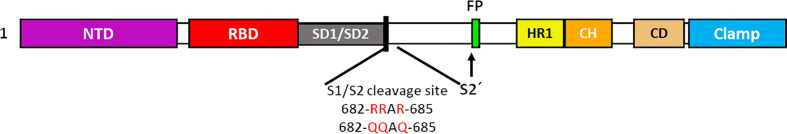
Medicago Inc.	Plant-based VLP / Co-VLP	VLP	IM (2)	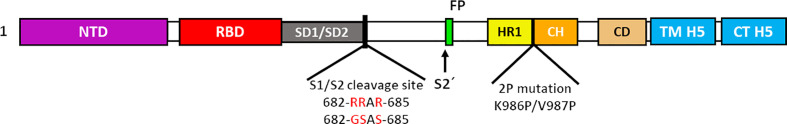
The University of Texas	HexaPro	PS	ND	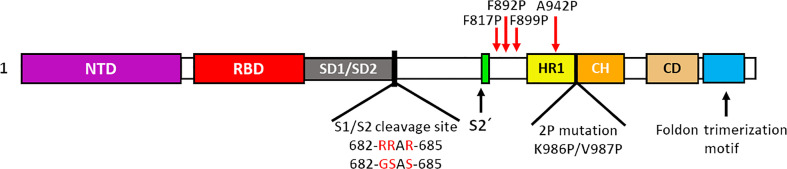
Stanford University	SΔC-Fer	VLP	ND	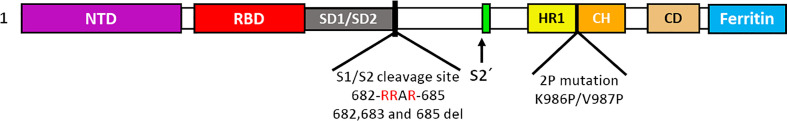

Vaccine platform acronym: nrW, non replicating viral vector; RNA, ribonucleic acid; DNA, desoxyribonucleic acid; PS, protein subunit; VLP, virus like particle. Route of administration. Oral, ND, no data; IM, intramuscular; ID, intradermal.

The company Vaxart has developed a vaccine that uses a nrVV adenovirus type-5 (Ad5), that encodes the complete S glycoprotein as it is expressed by the original SARS-CoV-2, preserving all the components of the S1 and S2 subunits (64). Similarly, the company Arcturus Therapeutics has used a complete S glycoprotein without modifications, however, they have opted for technologies of a self-replicating mRNA ready for translation (65). Other companies such as AstraZeneca and CanSino Biologics have developed nrVV such as AZD1222 and Ad5-nCOV. AZD1222 is based on a vector derived from a simian adenovirus ChAdOx-1 (66), and Ad5-nCOV (67) is based on the nrVV Ad5. Both technologies produce full-length S glycoprotein that contains the signal peptide of the human tissue plasminogen activator gene (tPA) at the N-terminus, with the aim of increasing expression in mammalian cells and which has been shown to improve humoral and cellular immune responses (66–68). Similarly, the Genexine consortium, has developed the GX19 vaccine, fusing tPA to the ectodomain of the S glycoprotein lacking the TM and CT, resulting in a soluble protein (69).

A series of vaccines employing S glycoproteins that include minimal changes have been tested with clinical success. The companies ReiThera, Leukocare and Univercells, using the non-replicative simian adenoviral vector GRAd32, have developed the GRAd-COV-2 vaccine, which contains the coding sequence of the full-length S glycoprotein, to which only an influenza HA tag (YPYDVPDYA) has been added, to facilitate the detection of antigen expression by commercially available HA antibodies (70). Similarly, the University of Munich, using the vector modified vaccinia virus Ankara (MVA), developed the MVA-SARS-2-S vaccine, with the same characteristics in the S glycoprotein, however, MVA-SARS-2-S encodes a stabilized S glycoprotein by replacing the K986P residues and V987P on top of the HR1 central helix of the S2 subunit (71). Pfizer-BioNTech have successfully developed the BNT162b2 mRNA vaccine, which also consists of the full-length S glycoprotein with the K986P and V987P mutation sites (72). The mRNA-1273 vaccine from the company Moderna contains the coding sequence for an S glycoprotein stabilized by a pair of proline substitutions (K986P/V987P), with a transmembrane anchor and an intact S1-S2 cleavage site (73). On the other hand, the INO-4800 vaccine, developed by Inovio Pharmaceuticals, The International Vaccine Institute and Advaccine (Suzhou) Biopharmaceutical Co., Ltd, have chosen to use a DNA vaccine in which the S glycoprotein is expressed with an IgE leader sequence, which has been documented to contribute to the improvement of expression in mammalian cells. In addition, the S glycoprotein is modified in the K986 and V987 sites by proline substitutions and thus achieve a stable S protein prefusion state (74).

When discussing the design of vaccines with S glycoproteins in a stable prefusion state, it is necessary to mention the modification to the furin cleavage site (682-RRARS-686), in which the substitution of arginine amino acid residues (R), by amino acids such as glutamine (Q), serine (S) or glycine (G), inhibit cleavage processing, and therefore, keep the S glycoprotein in a more stable prefusion state (75, 76). The Ad26.COV2.S vaccine, developed by the Janssen Pharmaceutical company, contains a full-length S glycoprotein stabilized by proline substitutions (K986P/V987P) removing the furin cleavage site by amino acid changes R682S and R685G (77). Similarly, the vaccine NVX-COV2373 from the Novavax company uses the substitution of prolines, as well as the modification of the furin cleavage site, substituting 3 arginines for glutamines (R682Q, R683Q and R685Q) (78, 79).

It is evident that the stability of the S glycoprotein is an extremely valuable property for the development of new vaccines against SARS-CoV-2, therefore, improving this property is essential for the development of more stable vaccine candidates. The SARS-CoV-2 vaccine SCB-2019 from Clover pharmaceuticals and SARS-CoV-2 Sclamp from CSL Ltd., share some characteristics with other companies previously mentioned, such as expressing exclusively the ectodomain of the S glycoprotein (80, 81), while deleting the signal sequence (SS) of the S glycoprotein. This vaccine uses a platform of subunit protein vaccines (SP) expressed in CHO cells. In addition, the vaccine SCB-2019 does not contain modifications in the furin cleavage site in S glycoprotein (81), keeping the composition of the ectodomain of the S glycoprotein intact (82). Both vaccines seek to improve the stability of the S glycoprotein, by adding trimerization domains at the C-terminal of the S glycoprotein, a trimer tag in the case of the SCB-2019 vaccine and a Clamp in the case of the Sclamp vaccine (81). The company Medicago Inc has developed the Co-VLP vaccine, based on the formation of VLPs, using the stabilized ectodomain of the S glycoprotein, with modifications to the furin site R682G, R683S and R685S and prolines substitution. The formation of VLPs was achieved by substituting the transmembrane domain and cytoplasmic tail of the original S glycoprotein for a transmembrane domain and cytoplasmic tail of influenza haemagglutinin 5 (83).

As we have mentioned, multiple research groups have developed vaccines based on stabilized forms of the S glycoprotein. HexaPro is a stabilized S containing four proline substitutions (F817P, A892P, A899P, A942P) as well as the two proline substitutions in S-2P (K986P/V987P), also incorporates mutations in the furin cleavage site (682-GSAS-685) and a C-terminal trimerization motif (84). HexaPro protein S turns out to be an ideal candidate for the development of new vaccines, due to its high stability, excellent expression and improved solubility, as well as preserved antigenicity (84, 85). Other research groups have proposed the rational design of vaccines using stabilized spike proteins with improved immunogenicity and antigenicity (86).

Some research groups have proposed the use of ferritin protein as a platform to develop vaccines (87). Ferritin is a protein that plays a key role in iron storage. Ferritin forms almost spherical nanoparticles (around 12 nm) by the self-assembly of 24 subunits (88). Among the important vaccine characteristics of ferritin protein are: robust immune response stimulation, thermal stability and adjuvant effect (87, 88). At Stanford University, two SARS-CoV-2 vaccine models have been developed based on H. pylori ferritin protein nanoparticles: (i) S glycoprotein full-length ectodomain (S-Fer, residues 1-1213) and (ii) S glycoprotein C-terminal 70 amino-acid deletion (SΔC-Fer, residues 1-1143), the two models fuse in their C-terminal to the ferritin protein but separated by a SGG linker. In addition to S glycoprotein, it contained a mutated furin cleavage site (RRAR for a single alanine) and two mutations at residues K986P/V987P, to stabilize the spike trimer. The SΔC-Fer vaccine showed better immunological effects (89). Methodologies combining RBD and ferritin nanoparticles are described in the next section.

Vaccine platform acronym: non replicating viral vector (nrVV), ribonucleic acid (RNA), desoxyribonucleic acid (DNA), protein subunit (PS), virus like particle (VLP). Route of administration. Oral, no data (ND), intramuscular (IM), intradermal (ID).

### RBD as Antigen for SARS-CoV-2 Vaccines

The S glycoprotein has been shown to be an excellent target for the development of vaccines against SARS-CoV-2; however, not all vaccines use the full-length S glycoprotein, some of them use highly immunogenic regions such as the RBD **(** **Table 2** **)**, which is found within the S1 subunit. RBM from RBD participates in the direct interaction with the ACE2 receptor (90). Some institutions such as The Jiangsu Provincial Center for Disease Control and Prevention, together with the Wantai BioPharm company, have chosen to develop RBD-based vaccines, such as the DelNS1-2019-nCoV-RBD-OPT1 intranasal vaccine, which consists of expressing RBD in an influenza rVV (91). Other companies such as Chongqing Boweibaitai Biopharmaceutical and Shanghai Bovax Biological Science & Technology, have applied for patent registration to protect vaccine candidates employing RBD with different lengths, particularly a RBD of 219 or 220 amino acids, produced in *Pichia pastoris* (92), an approach similar to that used in the development of vaccines against SARS-CoV-1. In this case, the mutation of glycosylation sites with RBDs of different lengths, induces robust antibody responses that resulted in the production of neutralizing epitopes, demonstrating that the loss of some glycosylation sites, can help improve protein stability (93–95).

**Table 2:** Strategies in the design of SARS-CoV-2 vaccines that use RBD of the S glycoprotein.

Developers	Vaccine	Vaccine platform	Phase	Design of vaccines using RBD as antigen
Jiangsu Provincial Center for Disease Control and Prevention	DelNS1-2019-nCoV-RBD-OPT1	VVr	Phase 2	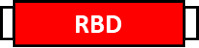
West China Hospital + Sichuan University	Recombinant (Sf9 cell)	PS	Phase 2	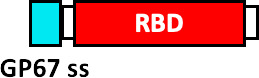
Guangzhou University of Chinese Medicine	ND	PS	ND	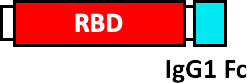
United Biomedical + COVAXX	UB-612	PS	Phase 1	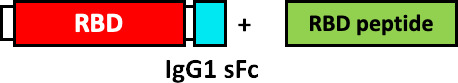
Kentucky BioProcessing	KBP-COVID-19	VLP	Phase 2	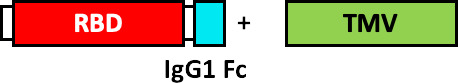
AdaptVac (PREVENT-nCoV)	ND	VLP	ND	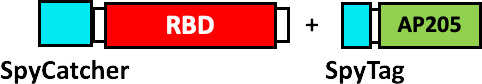
Anhui Zhifei Longcom Biopharmaceutical + Institute of Microbiology, Chinese Academy of Sciences	RBD-Dimer	PS	Phase 3	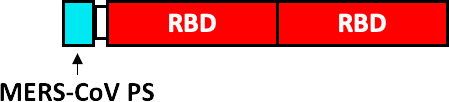
Pfizer/BioNTech	BNT162b1	RNA	Phase 3	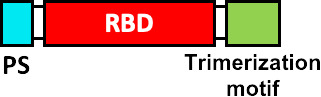
Cansino Biologics	ND	–	ND	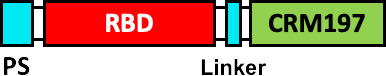
Sun Yat-sen University	ND	VLP	ND	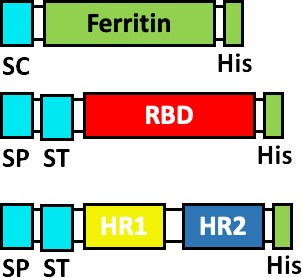

Envelope Glycoprotein Signal Sequence from Baculovirus GP67 (GP67 ss), crystallizable fragment of IgG1 (IgG1 Fc), Tobacco mosaic virus (TMV), coat protein of Acinetobacter phage AP205 (AP205), non-toxic mutant of diphtheria toxin (CRM197), SpyCatcher (SC), SpyTag (ST) and secretory signal peptide (SP).

The West China Hospital and the Sichuan University developed a vaccine produced by baculovirus in insect cells, which consists of RBD (aa 319-545), which incorporates a GP67 signal peptide at the N-terminal for the secretion of the fusion protein (GP67-RBD), demonstrating the robust generation of nAbs (96). Other institutions such as The Guangzhou University of Chinese Medicine, have applied for the protection of a patent consisting of a vaccine that comprises the RBD, fused to the Fc domain of IgG1, which has been shown to improve the recognition, absorption and processing of the antigen by the immune system cells (97).

The United Biomedical and COVAXX companies have developed the UB-612 vaccine produced in CHO cells, containing 8 components, including the RBD of the S glycoprotein (aa 331-530) fused to an Fc domain of Single chain IgG1, as well as 6 immunogenic peptides derived from highly conserved sequences from S, N and M proteins of SARS-CoV-1 and SARS-CoV-2, that stimulate T cell responses. The immunogenic peptides were modified with the addition of a Lys-Lys-Lys tail at the amino terminal end to improve its solubility and enrich its positive charge (98). More complex RBD-based vaccine designs have been developed by companies such as Kentucky BioProcessing, who have developed the PS KBP-COVID-19, based on RBD and produced in fast-growing tobacco plants (99). This vaccine contains RBD fused to the Fc domain of IgG1 through a linker (VEPKSCDKTHTCPPCP), and this fusion protein is finally conjugated with a tobacco mosaic virus (TMV) that functions as a VLP for the presentation of the antigen (100). Another VLP-based vaccine has been published by the company AdaptVac, as part of the PREVENT-nCoV consortium, who have developed a candidate vaccine using a SpyCatcher-RBD fusion protein, conjugated to a Spytag fused to the Acinetobacter phage coat protein (AP205), with the linker sequence (GSGTAGGGSGS). Furthermore, RBD was added with a binding protein (BiP) insect signal peptide at the N-terminus in addition to an EPEA (Glu-Pro-Glu-Ala) tag at the C-terminus. The proprietary peptide-binding Tag and a linker (GSGTAGGGSGS) were added to the N-terminus of the Acinetobacter phage AP205 coat protein (101).

Other companies have pursued different strategies. Anhui Zhifei Longcom Biopharmaceutical, and the Institute of Microbiology of the Chinese Academy of Sciences, have developed a universal betacoronavirus vaccine, including SARS-CoV-2, using the expression of a dimer RBD, aa R319-K537 from SARS-CoV-2, which consists of fusing in tandem a pair of RBDs under a signal peptide sequence from the S glycoprotein of MERS-CoV and expressing it on a commercial scale in CHO cells (102). The companies Pfizer and BioNTech have developed the BNT162b1 vaccine, which encodes the RBD of SARS-CoV-2 which is modified by the addition of a trimerization domain of the “fold” derived from T4 fibritin, to increase its immunogenicity, they have also added a signal sequence at the N-terminus of the RBD, to improve the efficiency of *in vivo* translation (103). On the other hand, Cansino Biologics has requested the protection of a vaccine that consists of a fusion protein that incorporates the RBD, a linker sequence (Gly Gly Gly Gly Ser) and a sequence encoding for the B subunit of the cholera toxin (CTB), or CRM197, a non-toxic mutant of diphtheria toxin (104). These proteins provide an adjuvant effect (105, 106) and induce a robust T-cell-dependent response (106).

At the Walter Reed Army Institute of Research, USA, four vaccine designs are proposed using the ferritin nanoparticle platform: (i) stabilized S-trimer-ferritin nanoparticles (SpFN, residues 12-1158), (ii) RBD-ferritin nanoparticles (RFN, residues 331-527), (iii) S1-ferritin nanoparticles (S1, residues 12-676), and (iv) RBD-NTD-ferritin nanoparticles (residues 331-527, 12-303). RBD-ferritin nanoparticles showed the highest levels of nAbs (107). On the other hand, the Institute of Human Virology, China, proposes nanoparticle vaccines that covalently conjugate 24 copies of RBD or RBD-HR protein subunits to the ferritin protein. They used *H. pylori* ferritin as the nanoparticle core. To conjugate the antigens on the surface of the ferritin nanoparticles they used the SpyTag/SpyCatcher system. The SpyTag (ST) was fused downstream from the secretory signal peptide (SP) at the N-terminus of RBD or HR (HR1/HR2). The SpyCatcher was fused to the ferritin protein sequence at the N-terminus. To the three genetic constructs (SC-Ferritin, ST-RBD and ST-HR) a 6 x His-tag was added at the C-terminus. Some ferritin nanoparticles were conjugated to RBD and other nanoparticles were conjugated to both RBD and HR. RBD and RBD-HR nanoparticles showed high neutralizing antibody titers, activation of the Th1 response and immunological memory (108).

Main Envelope Glycoprotein Signal Sequence from Baculovirus GP67 (GP67 ss), crystallizable fragment of IgG1 (IgG1 Fc), Tobacco mosaic virus (TMV), coat protein of *Acinetobacter phage* AP205 (AP205), non-toxic mutant of diphtheria toxin (CRM197), SpyCatcher (SC), SpyTag (ST) and secretory signal peptide (SP).

### Epitopes Derived From S Glycoprotein as Vaccine Antigens

**Figure 3:**
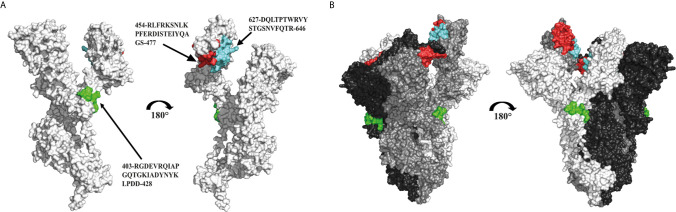
Immunogenic peptides used in a peptide vaccine against SARS-CoV-2. **(A)** Structural representation of a SARS-CoV-2 S glycoprotein monomer in closed state (PDB: 7DK3), showing the location of the epitopes and sequences used for the design of a peptide vaccine against SARS-CoV-2 (cyan, red and green). **(B)** Structural representation of the trimer formed by three protomers of the S glycoprotein (white, grey and black), and location of the antigenic epitopes in the oligomeric conformation.

The use of peptide fragments represents an attractive alternative for the development of new, safer vaccines with highly specific immune responses, avoiding allergenic and/or reactogenic consequences (109, 110). To combat the SARS-CoV-2 virus, some strategies proposed the design of peptide vaccines based on immunogenic epitopes of the S glycoprotein. The Federal Budgetary Research Institution State Research Center of Virology and Biotechnology Russia “Vector” has designed a peptide vaccine conjugated to a recombinant carrier protein and adsorbed on aluminum hydroxide. Synthetic peptides include: 403-RGDEVRQIAPGQTGKIADYNYKLPDD-248, 454-RLFRKSNLKPFERDISTEIYQAGS-477, 627-DQLTPTWRVYSTGSNVFQTR-646, 1192-NLNESLIDLQELGKYEQYIK-1211 and 1181-KEIDRLNEVAKNLNESLIDLQELGKYEQYIK-1211 of the SARS-CoV-2 S glycoprotein (RU0002738081), which are approved for use in Russia (111–113) (**Figure 3**).

UB-612 is another vaccine based on epitopes. This vaccine was developed for COVAXX/United Biomedical Inc. from Asia and is currently in phase 2 clinical trials (111). It is based on the S1-RBD peptides but incorporates cytotoxic T-lymphocyte (CTL)/Th epitopes which bind to human major histocompatibility complexes (MHC)-I and MHC-II for T-cell activation, respectively (114, 115). Five epitopes used in UB-612 are highly conserved in the sequences of the S, N and M proteins. It also contains patented technology, the UBITh^®^1, a peptide derived from the measles virus fusion protein (MVF) which function is to improve the immune response (98). The epitopes are mixed with CpG oligonucleotides to form immunostimulatory complexes that help stimulate the innate immune response *via* the toll-like receptor (TLR)-9 and improve the response of B and T cells. Finally, to complement the UB-612 formulation, the adjuvant aluminum phosphate is added to improve the Th2 response (98, 116, 117).

On the other hand, the University Hospital Tuebingen is developing a vaccine based on a multi-peptide cocktail of SARS−CoV−2 Human Leukocyte Antigen (HLA)−DR peptides. The vaccine called IMP (CoVac-1), is designed to be administered with the TLR 1/2 ligand XS15 and emulsified in the adjuvant Montanide ISA 51 VG (118, 119). HLA molecules are MHC from humans; therefore, HLA molecules are divided into class I (HLA-A, HLA-B, HLA-C) and class II (HLA-DR, HLA-DQ, HLA-DP). T lymphocytes recognize antigens bound to HLA and play a fundamental role in the response against viral infections (120). Nelde et al., characterized the HLA-DR peptides as potential T cell epitopes in SARS-CoV-2 infection. The SARS−CoV−2 HLA−DR peptides are used in the IMP (CoVac−1) vaccine (121). The use of adjuvants is also important to improve the immunogenicity of SARS−CoV−2 HLA−DR peptides. XS15 is an adjuvant with very important physical and functional characteristics such as: soluble in water, non-toxic and effective for the activation of peptide-specific CD4^+^ and CD8^+^ T lymphocytes (122). Meanwhile, Montanide ISA 51 VG is a water in oil emulsion based on a blend of mannide monooleate surfactant and mineral oil, this adjuvant increases antibody titers and CTL responses (123, 124).

Despite the numerous reverse vaccinology advances and all the bioinformatic tools that have been used in the analysis of the S glycoprotein and RBD domain sequences, few vaccines based on epitopes or immunogenic peptides have reached clinical phases **(** **Table 2** **)** (112, 125, 126). Reverse vaccinology refers to a methodology that uses bioinformatic tools to identify genomic sequences and structures of infectious agents for the design of vaccines *in silico* (127, 128). Reverse vaccinology has had an important impact on the development of vaccines for the current pandemic (SARS-CoV-2). The main advantages have been, the reduction of time and cost for the development of vaccines, it has helped to identify sequences of importance for coronaviruses that play a key role in infection. The identification of antigenic determinants facilitates the selection vaccine candidates without using the original pathogen (128–130). For example, Edison et al. (131) reports the use of reverse vaccinology and machine learning to predict SARS-CoV-2 vaccine candidates, including the S glycoprotein or non-structural proteins such as nsp3, 3CL-pro, and nsp8-10 (131).

## New Variants of SARS-CoV-2 and Effectiveness of S Glycoprotein-Based Vaccines

### New Variants of SARS-CoV-2

The emergence of new SARS-CoV-2 variants has made it clear that the end of the COVID-19 pandemic could come later than expected, due to the large number of new identified SARS-CoV-2 variants, some with risk potential to be more transmissible, virulent, pathogenic, or evade immunity induced by vaccination or previous infection (132, 133). In early March 2020, the first major SARS-CoV-2 variant was detected with the amino acid change D614G in the S glycoprotein. Before March 1, 2020, the D614G variant was found in 10% of 997 global sequences and by mid-May, it represented 78% of 12,194 sequences, suggesting a higher transmission rate (134, 135).

**Figure 4:**
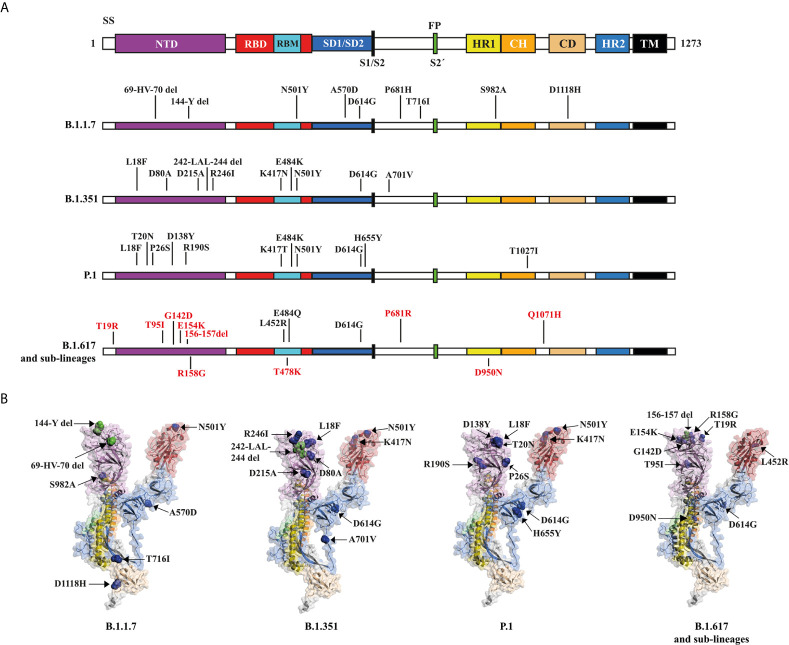
Structural mutations of the S glycoprotein in the SARS-CoV-2 variants: B.1.1.7., B.1.351, P.1, B.1.617 and his sub-lineages. **(A)** Schematic representation of the S glycoprotein (PDB:7DK3) and the changes present in variants. The amino acid mutations for the sub-lineages of B.1.617 are shown in red. **(B)** Structural representation of the S glycoprotein, showing deletion sites (del) (green dots) and mutation sites (blue dots) for each of the panel **(A)** variants. Some mutations are not shown because they are in unresolved regions.

During the COVID-19 pandemic, four variants of concern have attracted enough attention as a potential threat. The UK B.1.1.7 variant, B.1.351 of South African origin, P.1 originating in Brazil, and the Indian variant B.1.617 and his sub-lineages (B.1.617.1, B.1.617.2 and B.1.617.3), all of them with a series of relevant mutations in the S glycoprotein (136) (**Figure 4**).

In the United Kingdom, surveillance of new SARS-CoV-2 variants allowed the identification of variant B.1.1.7 in August 2020 (137, 138), having 17 mutations of which eight are in the S glycoprotein (139). Among the mutations that attract attention are N501Y in the RBM region, associated with an increased affinity for ACE2 (140). The P681H mutation, close to the furin cleavage site, a crucial region for infection and transmission (75, 139). The 69-HV-70 deletion, associated to immune escape in immunosuppressed patients and increased viral infectivity *in vitro*.

At the end of December 2020, a variant of South African origin was reported, named as B.1.351 (141). This variant has 10 mutations in the S glycoprotein, of which three are found in RBM: K417N, E484K and N501Y (142). K417N and E484K mutations have been shown to contribute to the escape of some nAbs (142). B.1.351 includes the D614G mutation, 242-LAL-244 deletions, and the R246I mutation in NTD, in addition to the A701V mutation near the furin cleavage site. Currently, variant B.1.351 has been reported in 58 countries, mostly South Africa and Europe, as well as the USA and Australia (142).

The variant P.1, a descendant of the Brazilian lineage B.1.1.28, was detected in late 2020 and early 2021. Variant P.1 contains 10 mutations in the S glycoprotein, in addition to D614G. Mutations include K417T, E484K, and N501Y in the RBD, L18F, T20N, P26S, D138Y, and R190S in the NTD, and H655Y near the furin cleavage site (143). Currently the P.1 variant has been detected in 26 countries, including countries in America and Europe (132).

At the end of 2020 and March 2021 in the Western state of Maharashtra, India, was identified the variant B.1.617 (144, 145) (**Figure 4**). B.1.617 has a pair of mutations, L452R and E484Q, which are of great concern due to its location in the RBD, and has been associated with improved inectivity (146), as well as greater affinity for ACE2 (147, 148). In addition to the above, the emergence of new sub-lineages could prolong the COVID-19 pandemic, since up to now 3 sub-lineages with high infectivity have been identified for B.1.617 (149).

### Spike Vaccine Effectiveness With SARS-CoV-2 Variants

Part of the success of SARS-CoV-2 vaccines depends on whether these vaccines will be able to protect against infection from the new SARS-CoV-2 variants. Until June 2021, 17 SARS-CoV-2 vaccines had been approved for emergency use in at least one country, while 35 were in phase 3 clinical trials. Of the 17 approved vaccines, 10 of them make use of the S glycoprotein or one of its immunogenic regions. The AZD1222 vaccine (nrVV) of AstraZeneca, employing a full length Spike glycoprotein without mutations, has been shown in clinical trials to be 74% (150) and 70.4% effective against variant B.1.1.7 (151), widely distributed in the UK. However, it is only 22% effective against the B.1.351 variant (152) and 10.4% effective on a second study (153).

The Pfizer-BioNTech and Moderna companies have achieved excellent results with the development of their BNT162b2 and mRNA-1273 vaccines, respectively. Both vaccines contain the coding sequence of a full-length S with the proline substitutions K986P/V987P. The clinical trials have shown the effectiveness of the BNT162b2 vaccine, achieving 95% protection against COVID-19 (154). This vaccine has shown to be 87% effective against B.1.1.7 and 72.1% effective against B.1.351 (155).

The vaccine NVX-CoV2373 (PS), developed by Novavax and Ad26.COV2.S vaccine (nrVV) of Janssen Pharmaceuticals, are vaccines that deploy the S glycoprotein with a mutated furin cleavage site and K986P/V987P proline substitutions. Novavax has opted for the expression of the Spike protein in insect cells using the recombinant baculovirus system, while Janssen Pharmaceutical is focusing on the use of an nrVV. In a recent clinical trial, NVX-CoV2373 has been shown to be between 95.6% (156) and 90.4% effective against the original strain from Wuhan, China (157), improving expectations, with early clinical trials reporting an effectiveness of 89% in the UK (158), which correlates with the 85.6% recently reported for the B.1.1.7 variant (156). In the case of the South African variant B.1.351, the first studies showed an effectiveness of 49% in South Africa (158) and more recently of 60% with variant B.1.351 (156). It is encouraging that the adenoviral vaccine Ad26COV2.S, developed by Janssen Pharmaceutical, reports a protective efficacy of 85% for the South African population (159).

So far, the only approved vaccine that uses Spike’s RBD, is the RBD-Dimer vaccine (or ZF2001), developed by the Anhui Zhifei Longcom Biopharmaceutical Company, who has obtained approval for emergency use in China and Uzbekistan (160). RBD-Dimer employs a pair of RBDs repeated in tandem under a signal sequence of MERS-CoV S glycoprotein expressed in CHO cells. RBD-Dimer proved to be well tolerated, with no serious adverse effects and very good immunogenicity, since after three doses neutralizing antibodies were detected in 95% of subjects immunized with 25 µg (161).

The only vaccine approved based on Spike epitopes is EpiVacCorona, with emergency use in Russia and Turkmenistan (160). Preclinical tests demonstrated the induction of antibodies against SARS-CoV-2 in 100% of immunized animals (162), however, clinical trials will be required to fully assess the effectiveness of this vaccine (163).

### Resistance of SARS-CoV-2 Variants to Current Vaccines

Understanding the immune response induced by SARS-CoV-2 vaccines or natural infections is essential to improve the preventive response against COVID-19 (164, 165). We are particularly motivated by the molecular and functional understanding of the antibody response against S glycoprotein, induced by vaccination or infection, which we recognize as a key factor to understand the efficacy of SARS-CoV-2 vaccines against the emergence of new variants and their resistance to neutralization by antibodies generated with current vaccines or natural infection. To date, at least 150 structures of nAbs targeting S glycoprotein from SARS-CoV-2 have been registered in the Protein Data Bank (PDB) (166).

**Figure 5:**
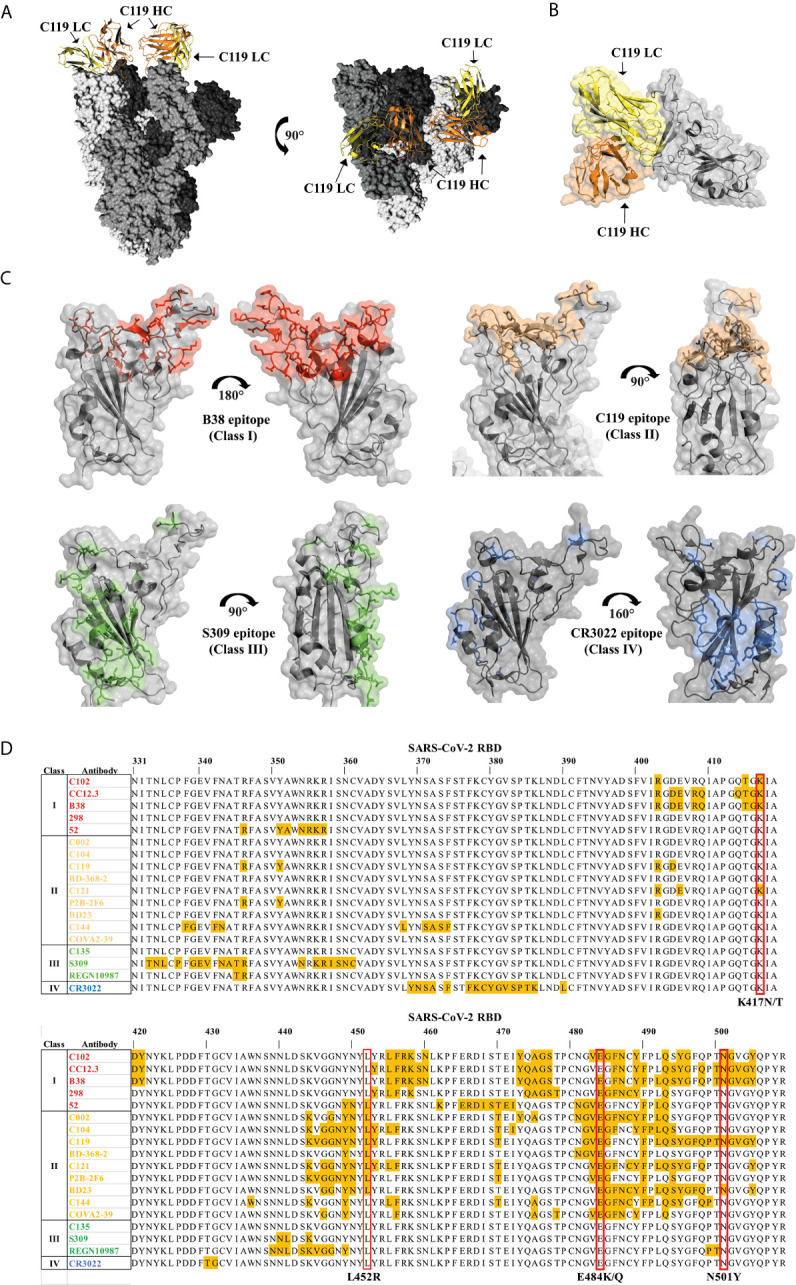
Association of neutralizing antibodies (nAbs) to RBD and mutations of variants B.1.1.7, B.1.351, P.1 and B.1.617 **(A)** General structure of the interaction of the nAb C119 blocking the binding site of ACE2 in RBD (PDB: 7K8W). **(B)** Interaction surface of the C119 antibody blocking the ACE2 binding site in RBD (PDB: 7K8W). **(C)** Regions in RBD that interact with nAbs of different classes. Class I: B38 Antibody (PDB: 7BZ5) (Red), C119 Antibody (PDB: 7K8W) (Sand), S309 Antibody (PDB: 7BEP) (Green), CR3022 Antibody (PDB: 6W41) (Blue). **(D)** RBD residues (yellow) interacting with class I, II, III and IV nAbs. The mutated amino acids of the variants B.1.1.7., B.1.351, P.1 and B.1.617 are highlighted in a red box.

Neutralization is regulated mainly by the non-covalent interaction of the amino acid residues of the antibody, present in the fragment antigen-binding (Fab), associated with epitopes present in the S glycoprotein domains (167–169), where the RBD is immunodominant and target of 90% of the neutralizing activity present in SARS-CoV-2 immune sera (170). Therefore, the nAbs that we review are exclusively associated with the RBD of the S glycoprotein, obtained from sera from convalescent patients (**Figure 5A**). Barnes (168) classifies the nAbs targeting RBD according to their association relationship: class 1) nAbs that block ACE2 binding site and that bind ‘up’ RBDs; class 2) nAbs that bind to both ‘up’ and ‘down’ RBDs, as well as contiguous RBDs; class 3) nAbs that bind outside the ACE2 binding site in RBDs in ‘up’ and ‘down’ conformation; and class 4) antibodies that bind ‘up’ RBDs without blocking ACE2 site (**Figures 5A, B**). Amino acid mutations in RBD can alter the binding affinity of nAbs and thereby contribute to resistance to neutralization. We review the amino acids in RBD that interact directly with nAbs, highlighting the mutations found in the RBD of variants B.1.1.7., B.1.351, P.1. and B.1.617 (**Figures 5C, D**).

Amino acid E484 appears to be a recurrent interaction site for class I, II and III nAbs that bind to the ACE2 binding domain in RBD, therefore the E484K mutation in the B.1.351 and P.1 variants, as well as E484Q in B.1.617 may be related to the reduction in the efficiency of nAbs (171–173). It has been observed that patients vaccinated with two doses of BNT162b2 vaccine (Pfizzer & Biontech), are capable of moderately neutralizing the new variants from SARS-CoV-2 containing the E484K mutation; however, the nAbs produced by a single dose of this vaccine seem to have less neutralizing effect (173).

Beltran et al. (174) have evaluated the neutralization potency of sera from individuals who received one or two doses of the BNT162b2 vaccine from Pfizzer or mRNA-1273 from Moderna, against pseudoviruses that present the S glycoprotein of 10 circulating variants, including B.1.1.7, B.1.351 and P.1. The results show that that variant B.1.1.7 has a slightly decreased neutralization which is dose-dependent; however, variants P.1 and B.1.351 present poor cross-neutralization, in which the number of doses does not seem to contribute to improving neutralization (174). On the other hand, other investigations suggest similar results for the BNT162b2 vaccine, showing good neutralization with variants such as B.1.1.7, but moderate against variant B.1.351 (175). The results obtained by Pfizer indicate small effects on neutralization associated with variant B.1.1.7 (del 69/70, N501Y and D614G), as well as B.1.351 (E484K + N501Y + D614G) (176). The company Moderna has also documented that its mRNA-1273 vaccine is less efficient in neutralizing the B.1.351 variant, but that such a reduction is not clinically significant (177).

The most recent emergence of variant B.1.617 and its sub-lineages has also cast doubt on whether the vaccines approved so far will be effective enough. Recent research shows that sera from convalescent patients or from individuals immunized with inactivated vaccines such as BBV152 (Covaxin) neutralize variant B.1.617 (145). However, sub-lineages such as B.1.617.1 turn out to be even 6.8 times more resistant to neutralization by sera from convalescent individuals vaccinated with mRNA-1273 from Moderna or BNT162b2 from the companies Pfizer-BioNTech, despite resisting the neutralization, most sera from convalescent patients and sera from all vaccinated patients neutralized variant B.1.617.1 (178). A similar study demonstrated that variant B.1.617 was partially resistant to neutralization by sera from convalescent patients and antibodies induced by BNT162b2 and mRNA-1273 vaccines (179). These investigations show that variant B.1.617 and sub-lineages are capable of resisting neutralization induced by vaccines that display the S glycoprotein. However, they appear to be efficient enough to protect individuals against reinfection.

It has been observed that a higher antibody titer allows the presence of nAbs directed to subdominant epitopes efficient enough to neutralize the SARS-CoV-2 variants containing E484K (171, 173). On the other hand, the N501Y mutation does not appear to compromise post-vaccination neutralization or infection (180). Nevertheless, variants carrying this mutation are associated with increased infectivity and virulence of SARS-CoV-2 (180). Meanwhile, the K417N/T mutation has been associated to a lesser degree with resistance to neutralization only for some antibodies (175, 180, 181).

The use of vaccines to prevent SARS-CoV-2 infection is probably the best strategy to combat SARS-CoV-2; however, it will also be important to identify whether the immunity induced in individuals previously infected with SARS-CoV-2 will be sufficient to protect against reinfection with SARS-CoV-2 variants. Some variants containing only mutations such as D614G have shown effective cross-neutralization with sera from individuals previously infected with SARS-CoV-2 wild type (134, 182, 183). Some research indicates that sera from convalescent patients is less efficient in cross-neutralizing variant B.1.1.7 (172, 184). On the other hand, variants carrying the E484K mutation such as B.1.351 and P.1 have been shown to escape monoclonal antibodies and avoid serum antibodies (172). In addition, the P.1 variant has been frequently identified in cases of reinfection by SARS-CoV-2 (185).

Variant B.1.351 containing the K417N, E484K and N501Y mutations in the RBD, coupled with other mutations in the S glycoprotein, is not efficiently neutralized by sera from patients recovered from SARS-CoV-2 infection (186), which could contribute significantly to the reinfection of individuals previously infected with the original SARS-CoV-2 strain from Wuhan.

Regarding variant B.1.617, it has been observed that the L452R mutation could contribute to an increase viral load (146) and increased infectivity (147, 148). On the other hand, the effects of E484Q mutation are poorly understood. However, the presence of both L452R and E484Q mutations have been associated to the decrease in the neutralization capacity by some nAbs (144). The B.1.617.1 sub-lineage containing the L452R, E484Q and P681R mutations has been reported to mediate entry into cells with slightly reduced efficiency compared to the original strain (187).

## Discussion

For the adequate design of COVID-19 vaccines, we must consider the selection of antigens, the proper selection of the platform for antigen production, adjuvants and other substances used during vaccine formulation. However, the success of the SARS-CoV-2 vaccines will also depend to a large extent on the production capacity to meet the volumes required by a global pandemic, an adequate distribution of doses, correct administration regimens, as well as appropriate use according to the type of vaccine, which could contribute to combat SARS-CoV-2 emerging variants.

Because the generation of new variants is aleatory, the longer the pandemic lasts, the higher the probability for the generation of new variants. Therefore, controlling rapidly the pandemic worldwide by a combination of a well-organized vaccination campaign coupled with strict sanitary measurements are key factors to prevent extending the pandemic for another year or even longer.

The S glycoprotein has been the main target for the development of vaccines against SARS-CoV-2 (4) and has been shown to be efficient enough to protect individuals who are vaccinated with this antigen or highly immunogenic regions of the same, such as the RBD (188). Companies such as Pfizzer & Biontech, AstraZeneca, Novavax, CanSino Biological, and Inovio Pharmaceuticals, have chosen to use the complete S glycoprotein, each following different strategies modifying the structural characteristics of the S glycoprotein, with the aim of improving its expression, recognition, stability or immunogenicity (67, 74, 78, 79, 150). Alternatively, other companies or academic institutions have chosen to use highly immunogenic regions within the S glycoprotein, such as the RBD. RBD-based vaccines may present modifications that improve their presentation, including the incorporation into VLPs (101), trimerization motifs (103), domain duplications (102) and even fusion carrier proteins (104). In addition, other types of vaccines such as those based on epitopes from the S glycoprotein are currently tested in animals models (112).

The wide variety of proposals approved and in clinical phases allows several options to design the best vaccination strategies against the SARS-CoV-2 (6). However, the emergence of new variants casts doubts on the effectiveness of the vaccines developed so far (174). It has been shown that some mutations present in new variants escape neutralization, particularly mutations found around the RBD (171–173). Given these mutations in the RBD, some vaccines have shown moderate neutralizing effects with variants such as B.1.351 and P.1 (150, 152, 158, 159). So far, only vaccines based on full-length S glycoprotein have been evaluated regarding their ability to neutralize new SARS-CoV-2 variants (182). It is imperative to evaluate the effectiveness of all vaccines against new variants if we intend to control the pandemic and prevent COVID-19 from becoming an endemic disease. Still, much like influenza, we cannot discard the possibility of having to generate new vaccines against emerging SARS-CoV-2 variants every year and implement frequent vaccination programs.

We consider that due to the emergence of new SARS-CoV-2 variants in countries with limited access to vaccines, the redesign SARS-CoV-2 vaccines based on these new variants is highly recommended.

We propose that the redesign of SARS-CoV-2 vaccines based on S glycoprotein, RBD or its epitopes should be considered for their proper use according to the geographic distribution of the SARS-CoV-2 variants. For example, epitope-based vaccines could have a lesser effect in countries with high prevalence of variants with immune escape, such as that observed with variants B.1.351 and P.1.

We should take full advantage of strategies such as genomic databases, structure prediction systems, and predictors of antigenic determinants in the design of vaccines against emerging variants (189), which may be useful assets to predict conformational and linear epitopes that can be recognized by B lymphocytes (190, 191). Most of the vaccines in use or in more advanced clinical trials utilize the complete SARS-CoV-2 virus, the full-length S glycoprotein, the RBD domain or RNA encoding the S glycoprotein **(** **Tables 1** and **2** **).**

Biomaterials are defined as substances of synthetic or natural origin, which can interact with living systems, in which they can perform therapeutic or diagnostic functions in order to improve the quality of life of individuals (192). The use of biomaterials in the development of vaccines against SARS-CoV-2 has been widely extended including: VLPs, DNA, RNA, liposomes, viral vectors, among others **(** **Tables 1** and **2** **).** However, the use of biomaterials in combination with peptides and predicted epitopes has not been sufficiently explored. There are biomaterials that form nano and microstructures which have been shown efficacy as antigen delivery systems, as enhancers of the immune response (adjuvant) and as antigen stabilizers (193, 194). Polymers and copolymers (195, 196), self-assembling proteins and self-assembling peptides (SAPNs and SAPs, respectively) (197, 198), microneedles (199), metallic nanoparticles (199, 200) and carbon nanomaterials (200). All of the previous mentioned biomaterials should be extensively explored in new generation of SARS-CoV-2 vaccines.

## Conclusion

We must recognize that the design and development of efficient vaccines against SARS-CoV-2 is the best strategy to combat COVID-19, fortunately many of them have shown high efficiency. Nevertheless, the emergence of new variants can jeopardize the success achieved so far with vaccination. To reach total control of the COVID-19 pandemic, the combination of several strategies will be necessary, which include an adequate distribution of SARS-CoV-2 vaccines taking into consideration the geographical distribution of the variants, the re-designing or SARS-CoV-2 antigens as well as the use of other technological tools such as bioinformatics and the use of novel biomaterials.

## Author Contributions

DM-F, JZ-C, AC-R, SA-S, AS, and LV wrote the manuscript. DM-F and SA-S generated the figures and tables. All authors contributed to the article and approved the submitted version.

## Funding

This manuscript was funded by the grant IV200320 from the Dirección General de Asuntos del personal Académico (DGAPA) to LV.

## Conflict of Interest

The authors declare that the research was conducted in the absence of any commercial or financial relationships that could be construed as a potential conflict of interest.

## Abbreviations

ACE2, Angiotensin converting enzyme 2; ADE, Antibody-dependent enhancement; CTB, B subunit of the toxin of the cholera; BiP, Binding protein insect signal pepide; CH, Central helix; AP205, Coat protein of Acinetobacter phage AP205; CD, Connector domain; COVID-19, Coronavirus Disease 19; IgG1 Fc, Crystallizable fragment of IgG1; CT, Cytoplasmic tail; E, Envelope protein; Fab, Fragment antigen-binding; FP, Fusion peptide; GP67 ss, Glycoprotein Signal Sequence from Baculovirus GP67; HR1, Heptad repeat 1; HR2, Heptad repeat 2; HLA, Human Leukocyte Antigen; tPA, Human tissue plasminogen activator gene; ID, Intradermal; IM, Intramuscular; MHC, Major histocompatibility complexes; M, Membrane protein; MERS-CoV, Middle East respiratory syndrome coronavirus; nAbs, Neutralizing antibodies; nrVV, No replicative viral vectors; Nsp1-16, Non-structural proteins; CRM197, Non-toxic mutant of diphtheria toxin; NTD, N-terminal domain; N, Nucleocapsid protein; PDB, Protein Data Bank; SP, Protein vaccines; RBD, Receptor-binding domain; RBM, Receptor-binding motif; rVV, Replicative viral vectors; SARS-CoV-2, Severe acute respiratory syndrome coronavirus type 2; SS, Signal sequence; ssRNA, Single-stranded positive RNA; S, Spike; SD1 and SD2, Subdomains 1 and 2; S1 and S2, Subunits 1 and 2; TMV, Tobacco mosaic virus; TLR, Toll-like receptor; TM, Transmembrane domain; TMPRSS2, Transmembrane protease serine 2; MVA, Vector modified vaccinia virus Ankara; VLP, Virus like particles.
